# Functional changes in health during and after pregnancy in people with spinal cord injury—an international cohort study

**DOI:** 10.1177/1753495X251415470

**Published:** 2026-02-03

**Authors:** Claire Mazzia, Sarah Daisy Kosa, Anne Harris, Roopjit Sahi, Anne Berndl

**Affiliations:** 1 Temerty Faculty of Medicine, 7938 University of Toronto, Toronto, ON, Canada; 2 Faculty of Health Sciences, 62703 McMaster University, Hamilton, ON, Canada; 3 School of Occupational and Public Health, 7984 Toronto Metropolitan University, Toronto, ON, Canada; 4 Division of Maternal-Fetal Medicine, Department of Obstetrics and Gynecology, 71545 University of Colorado School of Medicine, Aurora, CO, USA; 5 Division of Maternal-Fetal Medicine, Department of Obstetrics and Gynaecology, 71545 Sunnybrook Health Sciences Centre, Toronto, ON, Canada

**Keywords:** Pregnancy, spinal cord injury, physical disability, functional health

## Abstract

**Objective:**

To evaluate functional changes in patients with spinal cord injuries (SCIs) during and after pregnancy.

**Method:**

This is part of an international observational questionnaire examining pregnancy outcomes of people with SCI. Parameters assessed included pain, fatigue and bladder function. Participants categorized functions as improved, no change, or worsened during pregnancy and based on longstanding differences following delivery. Descriptive analysis was used.

**Results:**

This analysis included 263 pregnancies beyond 20 weeks’ gestation. Overall level and types of injuries, fatigue (66.7%), bladder function (65.9%), and edema (42.3%) were the most commonly reported aspects of functional health that worsened during pregnancy.

**Conclusions:**

Worsening fatigue and bladder function are the most common symptoms during pregnancy. Recovery is common in the post-partum period; however, nearly one-third of people experience longstanding changes. This study may provide guidance for pre-pregnancy consultation and inform expectations of pregnant people with SCI and their health care providers.

## Introduction

Spinal cord injury (SCI) is a common disability globally and has a myriad of effects on the body. Motor and sensory deficits are more widely known outcomes of SCI, but the autonomic nervous system which controls respiratory, cardiovascular, urinary, gastrointestinal, and sexual functioning, is also often affected.^1^ The majority of the literature that exists studying people with SCI focuses on males.^2^ There is limited research addressing how these aspects of functional health are impacted in the recovery of people assigned female at birth, and the implications this has on quality of life in people who become pregnant after SCI.^3^ Recent studies have found that this population is at greater risk of developing complications during pregnancy, such as hospitalization, urinary tract infections, pyelonephritis, autonomic dysreflexia, and pre-term birth compared to pregnant people without SCI.^3^ However, it is unclear how pregnancy impacts the functional health of individuals with SCI during pregnancy and after birth. Aspects of health that directly impact function in this group include strength, fatigue, pain, spasticity, edema, bladder, bowel, and sexual function.^4^ There are complex physiological changes that commonly occur after SCI, which have downstream effects on life satisfaction and quality of life. Functional health may be further affected by the endocrine and anatomic changes that are required for the body to support a developing fetus in the context of pregnancy. Studying the experiences of people with SCI who have had a pregnancy will support clinical decision-making and pre-conception counseling in these groups as well as guide future research in this field.^1^ This sub-study does not directly assess quality of life or life satisfaction.

This study aimed to assess participant's perception of functional health changes during and after a pregnancy greater than 20 weeks’ gestation post-SCI.

## Materials and methods

This research is part of the SCI-UP study, a research ethics-approved, international observational questionnaire examining urogenital and reproductive health outcomes of people with SCI. An online questionnaire was sent to SCI organizations and posted on social media in SCI support groups for willing participants to provide information regarding their injury, daily life, bladder and bowel health, and pregnancy history. The recruitment rate was 85.4% (1056/1237), and the completion rate was 73.8% (780/1056). Questions pertaining to functional health were not mandatory and were not answered by all participants.

This analysis included 263 pregnancies beyond 20 weeks’ gestation. Parameters assessed included strength, spasticity, fatigue, autonomic dysreflexia, orthostatic hypotension, breathing, pain, edema, falls, sexual dysfunction, bladder changes, bowel changes, and change in use of mobility device. Participants categorized these functions as improved, no change, or worsened during pregnancy, and longstanding changes following delivery. Descriptive analysis was used.

## Results

**Figure 1.**
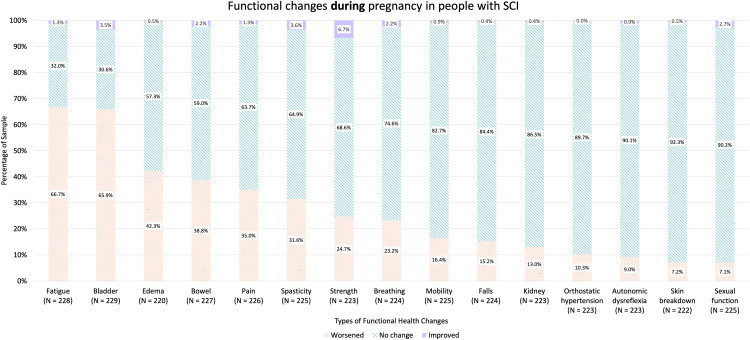
Stacked bar charts show the proportion of participants reporting worsened, no change, or improved function across multiple health domains during pregnancy. Percentages are shown within each bar, and sample sizes (*N*) are provided for each domain.

**Figure 2.**
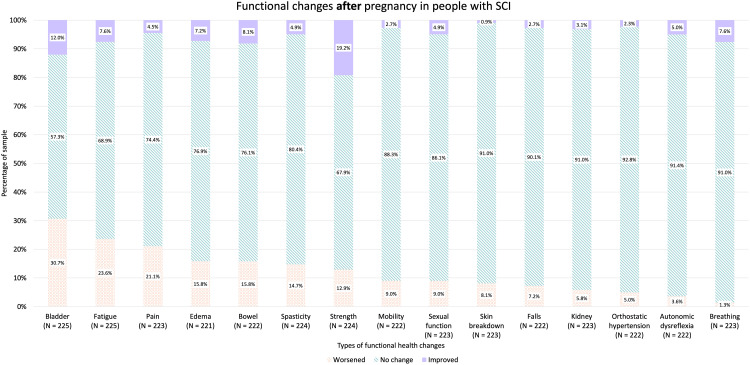
Stacked bar charts show the proportion of respondents reporting worsened, no change, or improved function across multiple health domains after pregnancy. Percentages are shown within each bar, and sample sizes (*N*) are provided for each domain.

In terms of demographics, 61.8% of participants reported residing in the USA, followed by 13.5% in Canada and 10.5% in Australia and New Zealand (Table 1). The thoracic level of SCI was most commonly observed (54.8%) followed by cervical SCI (31.6%) (Table 1). Participants most commonly reported having a complete ASIA-A injury (38.0%) followed by ASIA-B (22.8%) and ASIA-D injuries (19.3%) (Table 1). Participants with thoracic injury reported worsened bladder symptoms (64.5%), fatigue (61.8%), and edema (47.5%) during pregnancy; people with cervical injury had worsened fatigue (74.0%), bladder symptoms (61.6%), and bowel symptoms (45.2%) (Table 2). Worsened bladder symptoms predominated in lumbar (78.6%) and sacral level injuries (100%) (Table 2). Overall level and types of injuries, fatigue (66.7%), bladder function (65.9%), and edema (42.3%) were the most commonly reported aspects of functional health that worsened during pregnancy (Figure 1). In our sample, 61.2% pregnancies ended in a vaginal delivery, while 37.6% births occurred via caesarean delivery (Table 1). The mode of delivery for 1.1% were unreported (Table 1). Post-pregnancy, many people recovered back to baseline; however, worsened bladder function, fatigue, and pain persisted in 30.6%, 23.6%, and 21.1% of participants, respectively (Figure 2). More specifically, when looked at in separate groups according to level of injury, people with cervical and thoracic injuries as well as lumbar and sacral injuries all most commonly reported bladder function, pain, and fatigue worsened post-pregnancy compared to pre-pregnancy (Table 3). The prevalence of increased impairment in all three areas for people with cervical injuries was 23.9%, 22.2%, and 20.8%, respectively, while 27.6% of people with thoracic injuries experienced worsening bladder function, 20.5% reported increased fatigue, and 16.7% had increased pain after their pregnancy compared to pre-pregnancy (Table 3). Of people with lumbar and sacral injuries, 45.2% reported worsening bladder function, 41.9% reported increased fatigue, and 35.5% reported increased pain after a pregnancy relative to before (Table 3).

**Table 1.** Demographic information of participants.

Variable	Participants *N* = 171
Mean age at SCI	18.8 (±7.7)
Mean maternal age	28.8 (±5.5)
Country of residence	*N* (%)
USA	104 (61.8)
Canada	23 (13.5)
Australia and New Zealand	18 (10.5)
UK and Ireland	10 (5.8)
Spain	7 (4.1)
Other	9 (5.3)
Level of injury	*N* (%)
Cervical	58 (33.9)
Thoracic	88 (51.5)
Lumbar	22 (12.9)
Sacral	3 (1.8)
Severity of injury	*N* (%)
ASIA-A	65 (38.0)
ASIA-B	39 (22.8)
ASIA-C	30 (17.5)
ASIA-D	33 (19.3)
ASIA-E	3 (1.8)
Unreported	1 (0.6)
Variable	Pregnancies *N* = 263
Mode of delivery	*N* (%)
Vaginal delivery	161 (61.2)
Caesarean delivery	99 (37.6)
Unreported	3 (1.1)

**Table 2.** Functional changes by level of injury, most frequently reported as worsened during pregnancy.

Functional domains reported as worsened during pregnancy	Number of participants
Level of injury	*N* (%)
Cervical	
Fatigue	54/73 (74.0)
Bladder	45/73 (61.6)
Bowel	33/73 (45.2)
Thoracic	
Fatigue	76/123 (61.8)
Bladder	80/124 (64.5)
Edema	56/118 (47.5)
Lumbar and sacral	
Bladder	22/28 (78.6)
Fatigue	19/28 (67.9)
Pain	17/28 (60.7)

**Table 3.** Functional changes by level of injury, most frequently reported as worsened after pregnancy.

Functional domains reported as worsened after pregnancy	Number of participants
Level of injury	*N* (%)
Cervical	
Bladder	17/71 (74.0)
Pain	16/72 (61.6)
Fatigue	15/72 (45.2)
Thoracic	
Bladder	34/123 (27.6)
Pain	25/122 (20.5)
Fatigue	20/120 (16.7)
Lumbar and sacral	
Bladder	14/31 (45.2)
Fatigue	13/31 (42.0.8)
Pain	11/31 (35.5)

## Discussion

Understanding the physical impact of pregnancy on one's body and function both during pregnancy and after delivery are key pieces of information for people with SCI who are choosing pregnancy, and for health care professionals who counsel them pre-pregnancy, during pregnancy, and postpartum.

Based on this study, worsening bladder function during pregnancy is common for participants, independent of the level or severity of injury. However, the majority of participants recover back to baseline post-partum, with 31% having ongoing worse function. This is not outside the previously known rate of dysfunction of 15–45% in the postpartum period.^5^ In the non-SCI population, pelvic-floor muscle training has been found to be effective in decreasing the rate of urinary incontinence.^6^ Although there is minimal information regarding pelvic floor muscle training in people with SCI, it appears to have the potential to be beneficial to this population and may therefore be a possible therapy during pregnancy as well.^7^

Fatigue is a common complaint during pregnancy in the general population. SCI can also cause chronic fatigue, which likely has both physical and psychological components.^8^ The compounding effect of pregnancy in the context of SCI can thus lead to increased fatigue, and nearly 25% of participants in this study felt that their level of energy did not return completely after pregnancy, which is also seen in the non-SCI population associated with caring for a newborn. Physical rehabilitation to improve muscle strength, as well as social supports may be beneficial for new parents with SCI who are struggling with fatigue.

Chronic pain can be debilitating and may also contribute to fatigue. People with SCI often experience both musculoskeletal and neuropathic pain. Furthermore, pregnancy and childbirth are found to be risk factors for back pain in people without SCI.^9^ Advising patients that their pain may worsen during and after pregnancy and providing them with resources for pain management may help reduce the severity of their symptoms.

Conditions that are specific to people with SCI, such as spasticity, change in mobility device, skin break-down and autonomic dysreflexia, although impacted during the pregnancy, were less likely to persist following the pregnancy. This suggests that the kinds of residual physical impact of pregnancy may be similar between people with and without SCI.

Despite more people reporting worsening in symptoms during and post pregnancy, interestingly, 6.7% and 19.2% of people reported improved strength during and after their pregnancy, respectively (Figures 1 and 2). It is important to note that pregnancy can result in positive changes or benefits to health as well.

This study's strengths include the large and diverse population of people with SCI with a variety of levels and degrees of spinal cord lesions. Its weaknesses lie in having subjective description of change in function, as well as possible recall bias regarding function before, during, and after pregnancy. It is possible that other confounders, such as age, are impacting the reported changes in function that are not actually associated with the pregnancy. People who have a pregnancy without an SCI may have some changes in bladder and bowel function as well, so it is not known to what degree the reported changes are due to SCI vs due to pregnancy.

## Conclusion

This is the largest and most detailed study to date assessing important physical changes experienced by people with SCI during and after pregnancy. By understanding common impacts during pregnancy and those that tend to persist after birth, people with SCI and their caregivers can plan for and be prepared for any potential challenges. Although we can appreciate the changes experienced in pregnancy, we cannot know with certainty if the body changes experienced after pregnancy are attributed to the pregnancy or may be associated with another confounder, such as aging or the passage of time. A study using more standardized assessment of physical function, considering important confounders comparing people with SCI who have and have not had a pregnancy would be needed to further delineate the impact of pregnancy on these long-term differences. Future research could also look at possible modifiable risk factors for postpartum outcomes, such as trial of vaginal birth vs elective caesarean delivery while accounting for confounders such as age and pre-existing bladder and bowel dysfunction.
